# Developmental outcomes of young children with an autism diagnosis and its associated clinical correlates

**DOI:** 10.3389/fpsyt.2026.1836701

**Published:** 2026-06-15

**Authors:** Ramkumar Aishworiya, Erdembileg Sundarimaa, Siew-Pang Chan, Rakhi Vashishtha

**Affiliations:** 1 Child Development Unit, Khoo Teck Puat- National University Children’s Medical Institute, National University Hospital, Singapore, Singapore; 2 Department of Paediatrics, Yong Loo Lin School of Medicine, National University of Singapore, Singapore, Singapore; 3 Centre for Behavioural and Implementation Science Interventions (BISI), Yong Loo Lin School of Medicine, Singapore, Singapore; 4 Cardiovascular Research Institute, National University Heart Centre, Singapore, Singapore; 5 Institute of Geriatrics & Active Ageing, Tan Tock Seng Hospital, Singapore, Singapore

**Keywords:** autism, autism spectrum disorder, developmental outcomes, intervention, young children

## Abstract

**Introduction:**

Autism spectrum disorder is common in children with increasing prevalence, leading to efforts towards early identification and treatment initiation in young children. We aimed to identify the developmental trajectory of young children with an autism diagnosis and clinical factors associated with better developmental outcomes in a real-world setting.

**Methods:**

In this study, 38 children (mean age 21.9 ± 4.7 months) who were diagnosed at < 2 years of age with autism had developmental evaluations using the Mullen Scales of Early Learning done at baseline and after 1–2 years post diagnosis and intervention initiation.

**Results:**

In the sample, improvements in T-scores were observed for Receptive Language (mean 26.5 to 32.0, p=0.003) and Expressive Language (mean 26.0 to 30.7, p=0.010) at the group level, while Visual Reception, Fine Motor and overall Early Learning Composite did not change significantly at follow-up. Higher baseline T-scores were consistently associated with better follow-up scores across Fine Motor, Receptive Language, Expressive Language and Early Learning Composite domains. Age at initial presentation was significantly associated with Fine Motor outcomes (β = 1.26, p=0.031) while intervention hours were not independently associated with any developmental domain outcomes in the full sample. Among children with baseline developmental delay, higher intervention hours were associated with better follow-up scores in Receptive as well as Expressive Languages and Visual Reception; this was not seen for the entire sample.

**Conclusions:**

Our results support the role of early identification and intervention for autism; however, child-level characteristics influence developmental trajectories beyond intervention hours alone.

## 1 Introduction

Autism spectrum disorder (ASD or autism) is now identified at increasingly younger ages (1–5), driven by improvements in diagnostic pathways, greater community awareness, and the implementation of systematic screening tools such as the Modified Checklist for Autism in Toddlers, Revised with Follow-Up (M-CHAT-R/F) (6). These advances have meaningfully lowered the age of diagnosis and enabled children to access interventions earlier in development, which has previously been shown to lead to greater improvements in various aspects including language, social communication and adaptive skills (7–9).

Despite this progress, however, long−term outcomes in autism vary (10–15), with studies showing mixed results regarding who improves in terms of autism features and various developmental skills, who remain stable, and which developmental domains change most. The factors that account for these diverging trajectories are still not fully understood, including the roles of demographics (16), child characteristics and family context, as well as intervention-related factors such as type and hours of intervention (10). A recent meta-analysis by Sandbank et al. (2023) (17) found that naturalistic developmental behavioural interventions (NDBIs) led to statistically significant improvements on multiple autism features, such as behaviour and social communication. The same study however, noted that while behavioural interventions remain the most commonly recommended approach for autistic children in the United States, the strength of evidence supporting their effectiveness was mixed (17). Multiple studies have found that the amount of intervention provided in early childhood did not significantly predict developmental outcomes (10, 18), despite contrasting research stating the benefits of higher intervention intensity and greater improvements (19). Thus, the effects of intervention type and hours on the outcomes of autism symptoms remain mixed. Furthermore, results from studies conducted in a controlled research setting can differ from clinical settings where children can receive varying types and intensities of therapy depending on their context or circumstances (17, 20). Understanding outcomes in more real-life and naturalistic settings is critical, because they reflect what children actually receive and experience, and therefore provide a more holistic understanding of outcomes for children after an autism diagnosis.

Hence, the aim of this study is to examine real−world developmental outcomes among young children diagnosed with autism at a young age of < 2 years in a child development clinic. We aim to (1) describe the baseline characteristics and patterns of change in the developmental profiles of young children with an early autism diagnosis, (2) identify the effect of intervention hours on developmental outcomes, (3) identify other correlates of better developmental gains in these children over a 1 to 2-year period following diagnosis.

## 2 Materials and methods

### 2.1 Study sample

The study sample for this analysis was derived from a larger study (6) that assessed the performance of the M-CHAT-R/F in a community sample of young children aged around 18 months of age with consequent identification of a sub-set of children who were diagnosed with autism. Source participants for this study were children who were identified for further evaluation from the original larger study as they either had a positive screen (i.e., an at-risk screen defined as either high or moderate risk score) on the M-CHAT-R/F or had parental or primary care physician concerns for autism at 18 months of age.

The specific inclusion criteria for this study were children who: 1. were aged < 24 months old at time of baseline evaluation, 2. had a diagnosis of autism based on DSM-5 criteria following evaluation by a developmental behavioural paediatrician (DBP) and/or the Autism Diagnostic Observation Schedule (ADOS-2), toddler module and 3. had results of baseline evaluation and at least 1 follow-up evaluation in the clinic medical records.

The study proposal and procedures underwent review by the organisation’s ethical review board and deemed to be exempt from specific formal review due to the fully anonymous nature of data collection (NHG DSRB Ref: 2023/01002).

### 2.2 Study setting

This present study was a retrospective study based on medical records review of study participants who met the study inclusion criteria and who visited the Child Development Unit (CDU) at the research study site. The CDU provides family−centred, multidisciplinary assessment and intervention for children from birth to 7 years old with developmental, learning, and behavioural needs. The clinic is one of 2 national child development clinics and caters to the general population, providing diagnostic and therapeutic services to children with concerns for ASD and other neurodevelopmental disorders.

Intervention for children with a diagnosis of ASD was delivered in a 1–1 therapist to child format, at a frequency of 1 hour every 1 to 2 monthly. The content of therapy at the CDU was based on NDBIs using routine-based intervention for all children and delivered in a parent coaching format by experienced therapists (speech and language or occupational therapists). Provision and receipt of early intervention was based on real-world availability, compliance by the family and allocation; this was not dictated by the research team.

### 2.3 Study procedure

Medical information pertaining to the child’s baseline and follow-up evaluations, intervention-related data (i.e., hours of intervention) and family demographic data were extracted from the medical records system using a standardised data abstraction form.

Baseline evaluations for study participants included 1. Clinical interview and evaluation by a trained developmental behavioural paediatrician, and 2. the Mullen Scales of Early Learning (MSEL). The MSEL (21) is a widely used, standardized developmental assessment that provides direct evaluation of, language, and motor abilities and looks at overall development rather than just autism-related symptoms. The measure yields domain−specific T−scores across Visual Reception, Fine Motor, Receptive Language, and Expressive Language, as well as an Early Learning Composite (ELC) that reflects overall developmental functioning. The MSEL is well−validated in both clinical and research settings and is sensitive to developmental change in young children, including those with neurodevelopmental disorders. All assessments were administered by trained professionals with adequate reliability.

Subsequently, all study participants underwent standard clinical review at the CDU including early intervention as recommended by their DBP and subsequent serial MSEL examination (typically 1 year from baseline evaluations). These clinical reviews and MSEL assessments were scheduled and attended by the patient as part of routine care and were not mandated as study visits. The primary outcome of interest was the follow-up T-score for each MSEL domain (Visual Reception, Fine Motor, Receptive Language, Expressive Language) and the ELC.

In addition, information on child and family demographics, family socio-economic status based on income and parental education and clinical characteristics such as family history for autism, prematurity and presence of chronic medical conditions were also collected at baseline and extracted from medical records. Follow-up evaluations were conducted over a median of 13.5months (IQR 12.5–19.5 months; range 0.3–36.5 months), with the majority of children (52.6%) assessed between 12 and 18 months after baseline.

### 2.4 Statistical analysis

The final analytical sample included 38 participants who were assessed at two points. Baseline characteristics of the study participants were examined using basic descriptive statistics; mean (SD=) for continuous variables and n (%) for categorical variables.

To compare the baseline and follow-up scores for all MSEL domains, we first examined the normality distribution using skewness–kurtosis tests. All domains with approximately normal distribution were analysed using paired t-tests; domains with non-normal distribution were analysed using the Wilcoxon signed-rank test. Results are reported as mean (SD) for t-tests and median (IQR) for signed-rank tests. All tests were two-sided with α=0.05.

To examine the effects of total intervention hours on developmental outcomes, analysis of covariance (ANCOVA) models were employed with follow-up MSEL scores as the outcome and baseline T-scores included as a covariate in all models. Two models were conducted: a minimally adjusted model (Model 1) incorporating age at CDU presentation, gender and ethnicity as covariates, and a fully adjusted model (Model 2) which additionally included clinical factors, namely prematurity and family history of autism.

In addition to the above ANCOVA analyses, moderation analyses were conducted to examine whether baseline ELC delay modified the association between intervention hours and follow-up MSEL T-scores for each domain. As baseline domain-specific T-scores and overall ELC delay are inherently related constructs, variance inflation factors (VIF) were calculated for all predictors in the moderation models to assess for multicollinearity (22). VIF quantifies the degree to which the variance of a regression coefficient is inflated due to correlation with other predictors in the model, with values below 5 considered acceptable and values above 10 indicating serious concern. Given the small and unbalanced sample size, as well as potential heteroskedasticity, heteroskedasticity-robust (Huber–White) standard errors were used in all models.

## 3 Results

Based on baseline evaluations, 66 children were diagnosed with autism and eligible to be included in the study initially. Of these, 28 were lost to follow-up, resulting in a final analytical sample of 38 children who met study inclusion criteria. Among this final sample, 28 children had an ASD diagnosis made based on both clinical evaluation by a DBP and results of the ADOS-2 (among which the majority, N = 26 had ADOS-2 scores in the moderate-to-severe range) while 10 had an ASD diagnosis based on clinical evaluation by a DBP in accordance to DSM-5 criteria. Baseline characteristics of the study sample (N = 38) are presented in **Table 1**.

**Table 1:** Baseline characteristics of study sample (n=38).

Characteristics	Values
Age at CDU visit (months)	Mean 21.9 (SD = 4.7)
Age at intervention session (months)	Mean 23.8 (SD = 4.5)
Total intervention hours within follow-up period	Mean 9.2 hours (SD = 6.3)
Gender, n (%)	Boys = 30 (78.9%), Girls = 8 (21.0%)
Ethnicity, n (%)	Chinese = 21 (55.3%), non-Chinese = 17 (44.7%)
M-CHAT-R/F score	Mean 5.9 (SD = 3.0)
Mullen Scales of Early Learning Standard Scores	
– Visual Reception	Mean 35.1 (SD = 9.5)
– Fine Motor	Mean 35.9 (SD = 8.9)
– Receptive Language	Mean 26.5 (SD = 7.5)
– Expressive Language	Mean 26.0 (SD = 7.2)
Overall ELC	Mean 65.9 (SD = 11.8)
Screentime (weekday) (hours/wk)	Mean= 2.3 (SD = 1.8)
Screentime (weekend) (hours/wk)	Mean= 2.6 (SD = 2.0)
School attendance, n (%)	Not enrolled = 27 (71.1%), Full-day pre-school/childcare=9 (23.7%), Half-day pre-school/childcare=2 (5.3%)
Household income, n (%)	Below $2000 = 0, $2000-$4000 = 14 (36.8%), $4001-$6000 = 6 (15.8%), $6001-$8000 = 9 (23.7%), Above $8000 = 9 (23.7%)
Financial subsidies, n (%)	No = 31 (81.6%), Yes = 7 (18.4%)
Family history of autism=, n (%)	No = 34 (89.5%), Yes = 4 (10.5%)
Prematurity, n (%)	Yes = 4 (10.5%), No = 34 (89.5%)

The mean age at CDU visit was 21.9 months (SD = 4.7), while the mean age at the first intervention session was 23.8 months (SD = 4.5). Most were boys (n=30, 78.9%), predominantly of Chinese ethnicity (n=21, 55.3%). Caregiver-reported M-CHAT-R/F score averaged 6.0 (SD = 3.0). Most children were not enrolled in any childcare or day care (n= 27, 71.1%). Baseline MSEL standard score means were higher for non-language-based domains such as Visual Reception [35.1 (SD = 9.5)], and Fine Motor [35.9 (SD = 8.9)], compared to language domains, namely, Receptive Language 26.5 (SD = 7.5) and Expressive Language 26.0 (SD = 7.2). This also applied to baseline age equivalent MSEL scores, where mean for Visual Reception was 18.2 months (SD = 5.7) and Fine Motor 19.6 months (SD = 3.8) compared to language domains, namely, Receptive Language 14.2 months (SD = 5.4) and Expressive Language 12.6 months (SD = 5.7). Baseline mean overall ELC scores were 65.9 (SD 11.8) and majority of children (n=31, 81.6%) had a developmental delay at the baseline (i.e., ELC < 70). Reported screen time was ≤1 hour/day on weekdays among 12 children (32.4%), 1–2 hours/day among 10 children (27.0%) and >2 hours/day among 15 children (40.5%); weekend figures were similar with majority (48.7%) having > 2 hours/day of screen time. Mean weekday screen time was 2.3 hours/day (SD = 1.8), mean weekend screen time was 2.6 hours/day (SD-2.0). In terms of clinical attributes, most children did not have, any family history of autism or prematurity. In terms of socio-economic status, the sample had a spread of household income with a minority (n=7, 18.4%) receiving additional financial subsidies for healthcare suggesting a lower socio-economic status.

Across paired assessments, two of the four Mullen subdomains showed significant improvement in terms of T-scores. Receptive Language improved significantly from a mean of 26.5 (SD = 7.5) at baseline to 32.0 (SD = 13.3) at follow-up (p=0.003), and Expressive Language also improved significantly from a mean of 26.0 (SD = 7.2) to 30.7 (SD = 11.8) (p=0.010). (**Table 2**). Visual Reception scores did not show a statistically significant change over the follow-up period (median 34, IQR 30–40 at baseline vs median 33.5, IQR 24–41 at follow-up, p=0.68). Similarly, Fine Motor scores did not change significantly (mean 35.9, SD = 8.9 at baseline vs mean 33.7, SD = 12.8 at follow-up, p=0.33). Finally, the overall Early Learning Composite did not change significantly across the 2 time-points (mean 65.9, SD = 11.8 at baseline vs mean 69.7, SD = 20.6 at follow-up, p=0.18).

**Table 2:** Descriptive results for change in Mullen scores over time.

Domain	Baseline	Follow-up	Test used	p-value	Conclusion
Visual Reception	Median 34 (IQR 30–40)	Median 33.5 (IQR 24–41)	Wilcoxon signed-rank	p=0.68	No significant change
Fine Motor	Mean 35.9 (SD = 8.9)	Mean 33.7 (SD = 12.8)	Paired t-test	p=0.33	No significant change
Receptive Language	Mean 26.5 (SD = 7.5)	Mean 32.0 (SD = 13.3)	Paired t-test	p = 0.003	Significant improvement
Expressive Language	Mean 26.0 (SD = 7.2)	Mean 30.7 (SD = 11.8)	Paired t-test	p = 0.010	Significant improvement
Overall ELC	Mean 65.9 (SD = 11.8)	Mean 69.7 (SD = 20.6)	Paired t-test	p =0.18	No significant difference

ANCOVA models were conducted to identify significant factors that were associated with follow-up Mullen T-scores across each of the 4 developmental domains adjusting for baseline T-scores in all models. (**Table 3**).

**Table 3:** Results of ANCOVA analyses for each MSEL domain (n=37).

Developmental correlates	Model 1: minimally adjusted β (95% CI, p)	Model 2: fully adjusted β (95% CI, p)
Visual Reception		
CDU Therapy Hours	0.08 (-0.50–0.66, p=0.781)	-0.25 (-0.95–0.45, p=0.471)
Baseline Visual Reception T-score	0.16 (-0.49–0.81, p=0.621)	0.11 (-0.47–0.69, p=0.699)
Age at CDU visit (months)	0.83 (-0.31–1.97, p=0.149)	0.82 (-0.35–1.99, p=0.162)
Female Gender	1.20 (-13.08–15.47, p=0.865)	2.97 (-12.29–18.23, p=0.693)
Non-Chinese Ethnicity	4.98 (-4.05–13.99, p=0.269)	4.88 (-3.90–13.65, p=0.265)
Financial Subsidies	3.74 (-6.81–14.28, p=0.475)	-2.34 (-12.48–7.81, p=0.641)
Prematurity	—	7.13 (-5.14–19.40, p=0.244)
Family History of ASD	—	15.96 (-0.31–32.24, p=0.054)
Fine Motor		
CDU Therapy Hours	0.02 (-0.59–0.63, p=0.941)	-0.08 (-0.70–0.53, p=0.784)
Baseline Fine Motor T-score	0.50 (0.13–0.87, p=0.010)*	0.44 (0.07–0.82, p=0.021)*
Age at CDU visit (months)	1.39 (0.27–2.51, p=0.017)*	1.26 (0.13–2.39, p=0.031)*
Female Gender	-3.38 (-11.02–4.27, p=0.374)	-3.04 (-10.93–4.86, p=0.437)
Non-Chinese Ethnicity	3.66 (-3.89–11.21, p=0.331)	3.56 (-3.75–10.87, p=0.327)
Financial Subsidies	6.30 (-3.69–16.28, p=0.208)	4.08 (-6.99–15.14, p=0.457)
Prematurity	—	8.19 (-0.95–17.33, p=0.077)
Family History of ASD	—	5.10 (-10.48–20.69, p=0.508)
Receptive Language		
CDU Therapy Hours	0.04 (-0.58–0.65, p=0.908)	-0.15 (-0.86–0.56, p=0.673)
Baseline Receptive Language T-score	0.96 (0.40–1.52, p=0.001)*	0.85 (0.32–1.38, p=0.003)*
Age at CDU visit (months)	0.57 (-0.74–1.87, p=0.383)	0.55 (-0.86–1.95, p=0.434)
Female Gender	-4.72 (-12.42–2.99, p=0.221)	-3.22 (-10.96–4.53, p=0.403)
Non-Chinese Ethnicity	2.61 (-5.62–10.85, p=0.522)	2.55 (-5.45–10.55, p=0.519)
Financial Subsidies	2.56 (-6.75–11.88, p=0.578)	-0.84 (-11.44–9.76, p=0.873)
Prematurity	—	6.64 (-4.97–18.25, p=0.251)
Family History of ASD	—	8.64 (-7.67–24.95, p=0.287)
Expressive Language		
CDU Therapy Hours	0.28 (-0.15–0.72, p=0.195)	0.002 (-0.54–0.54, p=0.993)
Baseline Expressive Language T-score	0.66 (0.13–1.20, p=0.017)*	0.83 (0.28–1.37, p=0.004)*
Age at CDU visit (months)	0.67 (-0.14–1.48, p=0.103)	0.78 (-0.09–1.65, p=0.077)
Female Gender	3.69 (-6.81–14.20, p=0.478)	6.25 (-4.59–17.09, p=0.248)
Non-Chinese Ethnicity	4.74 (-2.62–12.11, p=0.198)	5.45 (-1.76–12.65, p=0.133)
Financial Subsidies	3.37 (-7.40–14.14, p=0.528)	-1.55 (-17.56–14.46, p=0.845)
Prematurity	—	-5.21 (-12.16–1.75, p=0.136)
Family History of ASD	—	13.84 (-3.42–31.10, p=0.112)
Early Learning Composite		
CDU Therapy Hours	0.11 (-0.67–0.89, p=0.778)	-0.29 (-1.14–0.56, p=0.486)
Baseline ELC score	0.75 (0.21–1.29, p=0.008)*	0.73 (0.29–1.18, p=0.002)*
Age at CDU visit (months)	1.55 (-0.15–3.25, p=0.073)	1.53 (-0.29–3.35, p=0.095)
Female Gender	0.66 (-16.28–17.61, p=0.937)	3.12 (-14.47–20.71, p=0.719)
Non-Chinese Ethnicity	3.50 (-8.08–15.08, p=0.542)	3.24 (-7.79–14.27, p=0.553)
Financial Subsidies	6.00 (-9.98–21.97, p=0.449)	-1.52 (-20.67–17.64, p=0.872)
Prematurity	—	7.76 (-8.33–23.86, p=0.332)
Family History of ASD	—	19.26 (-4.13–42.64, p=0.103)

*Statistically significant at p < 0.05; β, regression coefficient; CI, confidence interval; CDU, Child Development Unit; MSEL, Mullen Scales of Early Learning; ASD, Autism Spectrum Disorder.

For the Visual Reception domain, intervention hours, age at CDU visit, gender, ethnicity and financial subsidies were not significantly associated with follow-up Visual Reception T-scores in the minimally adjusted model (Model 1). These findings were consistent in the fully adjusted model (Model 2), with no significant associations observed for any covariate including CDU therapy hours, prematurity or family history of autism.

For the Fine Motor domain, higher baseline Fine Motor T-scores were significantly associated with better follow-up Fine Motor T-scores in both the minimally adjusted model (Model 1: β = 0.50, 95% CI: 0.13–0.87, p=0.010) and the fully adjusted model (Model 2: β = 0.44, 95% CI: 0.07–0.82, p=0.021), indicating that children with relatively better baseline Fine Motor functioning maintained that advantage at follow-up. With each additional month of age at CDU visit, which ranged between 16–36 months for our sample, children showed significantly better Fine Motor T-scores at follow-up in both Model 1 (β = 1.39, 95% CI: 0.27–2.51, p=0.017) and Model 2 (β = 1.26, 95% CI: 0.13–2.39, p=0.031). Intervention hours were not significantly associated with Fine Motor outcomes in either model. No other demographic or clinical factors were significantly associated with Fine Motor T-scores across adjusted models.

For the Receptive Language domain, higher baseline Receptive Language T-scores were significantly associated with better follow-up Receptive Language T-scores in both the minimally adjusted model (Model 1: β = 0.96, 95% CI: 0.40–1.52, p=0.001) and the fully adjusted model (Model 2: β = 0.85, 95% CI: 0.32–1.38, p=0.003), suggesting that children with relatively better baseline Receptive Language functioning maintained that advantage at follow-up. Intervention hours, age at CDU visit and all other demographic and clinical factors were not significantly associated with Receptive Language T-scores in either model.

For the Expressive Language domain, higher baseline Expressive Language T-scores were significantly associated with better follow-up Expressive Language T-scores in both the minimally adjusted model (Model 1: β = 0.66, 95% CI: 0.13–1.20, p=0.017) and the fully adjusted model (Model 2: β = 0.83, 95% CI: 0.28–1.37, p=0.004) consistent with findings in the Fine Motor and Receptive Language domains. Intervention hours, age at CDU visit and all other demographic and clinical factors were not significantly associated with Expressive Language T-scores in either model.

For the overall ELC, higher baseline ELC scores were significantly associated with better follow-up ELC scores in both the minimally adjusted model (Model 1: β = 0.75, 95% CI: 0.21–1.29, p=0.008) and the fully adjusted model (Model 2: β = 0.73, 95% CI: 0.29–1.18, p=0.002), consistent with the pattern observed across other domains. Age at CDU visit showed a trend towards significance in both models but did not reach statistical significance (Model 1: β = 1.55, 95% CI: −0.15–3.25, p=0.073; Model 2: β = 1.53, 95% CI: −0.29–3.35, p=0.095). Intervention therapy hours and all other demographic and clinical factors were not significantly associated with ELC at follow-up in either model.

Moderation analyses were conducted to examine whether baseline ELC delay (ELC ≤70) modified the associations between intervention hours and follow-up MSEL T- scores across developmental domains adjusting for baseline T-scores and age at CDU visit in all models. Assessment of VIF confirmed no problematic multicollinearity among predictors, with VIF values ranging from 1.5 to 1.9 for baseline domain T-scores and 3.0 to 4.3 for baseline ELC delay, all within acceptable thresholds. The findings are presented in **Table 4**. For the Visual Reception domain, baseline ELC delay was significantly associated with lower follow-up Visual Reception T-scores (β = −25.53, p=0.001; 95% CI: −39.21 to −11.85), indicating that children with baseline developmental delay had significantly lower Visual Reception scores at follow-up compared to those without delay. A significant interaction was observed between baseline ELC delay and intervention hours (β = 1.35, p=0.010; 95% CI: 0.35–2.34), indicating that among children with baseline developmental delay, greater intervention hours were associated with significantly better Visual Reception T-scores at follow-up. For Receptive Language, a significant interaction between baseline ELC delay and intervention hours was also observed (β = 1.61, p=0.005; 95% CI: 0.52–2.70), indicating that only among children with baseline developmental delay, greater intervention hours were associated with significantly better Receptive Language T-scores at follow-up. This finding is consistent with the primary models, in which intervention hours were not significantly associated with Receptive Language outcomes in the full sample. A significant interaction between baseline ELC delay and intervention hours was also observed for Expressive Language (β = 1.53, p<0.001; 95% CI: 0.87–2.19), indicating that among children with baseline developmental delay, greater intervention hours were associated with significantly better Expressive Language T-scores at follow-up. In contrast, no significant moderation effect was observed for Fine Motor outcomes, as the interaction term between baseline ELC delay and intervention hours did not reach statistical significance (β = 0.44, p=0.356; 95% CI: −0.52–1.41).

**Table 4:** Moderation analyses results examining whether baseline ELC delay modified the association between CDU therapy hours and follow-up MSEL T-scores (n=37).

Variable	β (95% CI, p-value)
Visual Reception (n=37)	
Baseline Visual Reception T-score	-0.04 (-0.70–0.63, p=0.910)
CDU Therapy Hours	-0.95 (-1.56– -0.34, p=0.003)*
Baseline ELC delay (≤70)	-25.53 (-39.21– -11.85, p=0.001)*
CDU Hours × ELC delay (≤70)	1.35 (0.35–2.34, p=0.010)*
Age at CDU visit (months)	0.68 (-0.10–1.46, p=0.087)
Fine Motor (n=37)	
Baseline Fine Motor T-score	0.48 (0.09–0.88, p=0.018)*
CDU Therapy Hours	-0.27 (-0.76–0.22, p=0.271)
Baseline ELC delay (≤70)	-7.93 (-21.02–5.16, p=0.226)
CDU Hours × ELC delay (≤70)	0.44 (-0.52–1.41, p=0.356)
Age at CDU visit (months)	1.32 (0.33–2.30, p=0.011)*
Receptive Language (n=37)	
Baseline Receptive Language T-score	0.73 (0.08–1.38, p=0.030)*
CDU Therapy Hours	-1.18 (-1.98– -0.38, p=0.005)*
Baseline ELC delay (≤70)	-16.78 (-36.14–2.59, p=0.087)
CDU Hours × ELC delay (≤70)	1.61 (0.52–2.70, p=0.005)*
Age at CDU visit (months)	0.56 (-0.57–1.69, p=0.319)
Expressive Language (n=37)	
Baseline Expressive Language T-score	0.44 (-0.11–0.99, p=0.110)
CDU Therapy Hours	-0.92 (-1.44– -0.40, p=0.001)*
Baseline ELC delay (≤70)	-22.85 (-35.01– -10.69, p=0.001)*
CDU Hours × ELC delay (≤70)	1.53 (0.87–2.19, p<0.001)*
Age at CDU visit (months)	0.52 (-0.22–1.25, p=0.161)

*Statistically significant at p < 0.05. β = regression coefficient; CI, confidence interval; CDU, Child Development Unit; ELC, Early Learning Composite; MSEL, Mullen Scales of Early Learning.

Baseline ELC delay defined as ELC ≤70 at baseline assessment. CDU Hours × ELC delay = interaction term between CDU therapy hours and baseline ELC delay.

## 4 Discussion

This study examined real-world developmental outcomes among young children diagnosed with autism before the age of two years in a tertiary clinical setting, with the aims of describing developmental trajectories, evaluating the effect of intervention exposure, and identifying correlates of developmental change over a two-year follow-up period. The results from our study indicated that significant improvements in T-scores were observed in two of the four developmental domains — Receptive Language and Expressive Language, between baseline and follow-up, while Visual Reception and Fine Motor did not show statistically significant change, likely due to measurement constraints at the lower end of the T-score scale in this population, whereas overall developmental functioning, as measured by the ELC, remained stable at the group level. Furthermore, intervention hours were not independently associated with developmental outcomes in adjusted models across domains, aligning with prior mixed evidence regarding the role of intervention intensity in early autism outcomes. Age at CDU presentation was significantly associated with Fine Motor outcomes across both adjusted models, while higher baseline T-scores were consistently associated with better follow-up scores across Fine Motor, Receptive Language, Expressive Language and ELC domains. Other demographic and clinical factors showed limited or non-significant associations. Notably, our results also suggest that, among the subset of children with developmental delay at baseline, higher intervention hours were associated with greater follow-up scores in three domains — Visual Reception, Receptive Language and Expressive Language — representing an expansion of this finding compared to the original analysis. Together, these findings highlight substantial domain-specific developmental gains following early autism diagnosis and intervention in a real-world clinical context, while underscoring the importance of child-level characteristics in shaping developmental trajectories beyond intervention dose alone.

Our findings suggest that among the developmental domains examined, Fine Motor outcomes were significantly associated with age at CDU presentation, with each additional month of age associated with better Fine Motor T-scores at follow-up across both adjusted models. This may be indirectly related to these children having less autism features and/or developmental delays, thereby leading to a later age of evaluation, although we did not specifically measure degree of autism features. Indeed, higher baseline functioning was associated with greater follow-up scores for some of the developmental domains across both adjusted models in our sample as well. This is also consistent with findings from a recent large meta-analysis that showed better baseline developmental skills and lower autism features to be associated with greater post-intervention effects (23). However, this association should be interpreted cautiously and does not imply that older age at presentation confers a developmental advantage; rather it is likely the baseline developmental status (i.e. presence of and degree of developmental delay at baseline) that is more pertinent for follow-up developmental status. Rather that the child’s fine motor skills were better at an older age. Nonetheless, it is pertinent to note that Receptive Language and Expressive Language T-scores improved significantly at the group level, indicating that meaningful developmental gains in language domains were observed following early diagnosis and intervention. Therefore, these findings support the continued role for early diagnosis and intervention in young children with autism.

In our sample, as a group, the overall Early Learning Compositive score (which is representative of overall development) did not improve significantly over time suggesting that despite the progress in individual domains, overall developmental status did not necessarily change. This could be related to the relatively short time span of follow-up in this sample as developmental changes need longer time to manifest typically. Further, this group-level finding is also likely related to baseline development, with a differential effect on subsequent improvement over time. This is supported by results of the moderation analysis, which showed that among those with lower baseline development, greater intervention hours were associated with greater follow-up T-scores in Receptive Language, Expressive Language and Visual Reception- three of the four domain examined. Although not replicated at the group level in our sample, this positive effect of intervention hours was also noted in the above-mentioned meta-analysis, although other studies (10, 18) have suggested that overall intervention hours did not make a difference in everyone, as per our findings. This has clinical implications – these children with greater delays at baseline may benefit more from intervention and hence potentially be stratified to receive more intervention. This is especially pertinent in the real-world context with limited resources and long wait times for autism-specific intervention. Further, this supports arguments towards early identification of children with autism and treatment initiation, through measures such as systematic screening. Screening in early childhood typically identifies children who present with more autism features and have existing developmental delays (24, 25) – this is the group in whom greater intervention associated with higher developmental scores for three of the four domains based on our results.

Strengths of this study include the use of data from a real-world clinical context reflective of the typical intervention received within a naturalistic setting as opposed to control research-cohorts where intervention is often at higher frequency. We also add to the literature by examining this research question using a Southeast Asian sample which is under-represented in global autism research. However, the study does have several limitations. First, we had a small sample size which limits the generalizability and interpretation of findings. This also gave us limited power for additional subgroup analysis including based on family socio-economic status and/or parental education for example. Future studies will involve larger cohorts of children to allow for more robust evaluations. Secondly, the significant loss to follow-up among children initially diagnosed with autism (42.4%) This is a major limitation that warrants explicit acknowledgement. Of the 66 children initially diagnosed with autism, only 38 had complete follow-up data. Those who did not return for follow-up evaluation may have differed systematically from those who did. Children with better early developmental trajectories may have disengaged from clinical follow-up as families perceived less need for continued monitoring, which would mean the study sample is enriched for children with more persistent difficulties and greater severity. Conversely, families facing greater socioeconomic burden or logistical challenges may have been less able to attend follow-up, introducing a different form of bias. The direction of this attrition bias is uncertain, but either scenario limits the representativeness of the analytical sample and findings should be interpreted with this in mind. Third, the retrospective design, with data collected from existing medical records rather than through a prospectively designed protocol, may have introduced additional selection bias and residual confounding, limiting causal inference; this also resulted in a range of follow-up period among the sample. Additionally, while the real-world setting of this study enhances its ecological validity and reflects the heterogeneity of clinical practice, the provision and receipt of early intervention — both at the CDU was determined by real-world availability, family compliance and resource allocation rather than by a standardized research protocol. This limits the degree to which intervention intensity and type could be controlled or systematically characterised and should be considered when interpreting the associations observed between intervention exposure and developmental outcomes. Lastly, the study sample constituted children with an early autism diagnosis before 2 years of age at a single developmental clinic this may have resulted in a sample with more severe symptomatology and/or with co-existing developmental This limits the generalizability of our findings to the broader autism population but is still useful in terms of insights into this specific subset of children who typically are the recipients of early childhood autism intervention.

### 4.1 Conclusions

This pilot study examining developmental trajectories of very young children diagnosed with autism shows that significant improvements in Receptive Language and Expressive Language T-scores were observed over time, while Visual Reception, Fine Motor and overall Early Learning Composite scores did not show statistically significant change. However, intervention hours are not directly associated with degree of developmental gains although for those children who have developmental delays at baseline, a greater number of intervention hours is associated with improved scores in Expressive Language, Receptive Language and Visual Reception domains. Our findings support the overall motion towards early identification and treatment initiation in autism and the role of early intervention towards better developmental outcomes.

## Data Availability

The raw data supporting the conclusions of this article will be made available by the authors, without undue reservation.
